# IRF1 ameliorates synaptic dysfunction through the modulation of O-GlcNAcylation on GluN1 subunit of NMDAR

**DOI:** 10.1186/s13195-025-01857-w

**Published:** 2025-09-30

**Authors:** Xing Fan, Hao Wang, Cuiping Guo, Shijia Huang, Liye Xia, Zheng Zhou, Ran Tao, Mingzhe Li, Xiaochuan Wang, Wei Qian

**Affiliations:** 1 Department of Biochemistry and Molecular Biology, School of Medicine, Key Laboratory of Neuroregeneration of Jiangsu and Ministry of Education, Co-innovation Center of Neuroregeneration, Nantong University, Nantong, 226001 China; 2 Hubei Key Laboratory of Cognitive and Affective Disorders, School of Medicine, Jianghan University, Wuhan, 430056 China; 3 Guangzhou National Laboratory, Guangzhou International Bio Island, No. 9 XingDaoHuanBei Road, Guangzhou, 510005 China; 4 Affiliated Hospital of Nantong University, Medical School of Nantong University, Nantong, 226001 China; 5 School of Language, Culture and Society, University of Leeds, Woodhouse, Leeds, LS2 9JT England

**Keywords:** IRF-1, O-GlcNAcylation, GluN1, Synaptic dysfunction, Alzheimer’s disease

## Abstract

**Background:**

Synaptic dysfunction, which occurs before the formation of amyloid plaques (Aβ) and neurofibrillary tangles (NFTs), is strongly associated with cognitive deficits and represents major early clinical features of Alzheimer’s disease (AD). Abnormal NMDAR signaling emerges as a noticeable feature of synaptic dysfunctions in AD. Nonetheless, the underlying mechanisms of NMDAR dysfunctions remain unclear.

**Methods:**

3xTg-AD mice were injected with AAV-IRF1. Cognitive function was assessed using behavioral tests, while biochemical and immunofluorescence analyses were conducted to evaluate the protein levels of IRF-1, OGA, subunits of NMDAR, O-GlcNAcylation of NMDAR subunits, and internalization of NMDA receptors. Synaptic alterations in the hippocampus were detected by electrophysiology and Golgi staining.

**Results:**

In the present study, we demonstrate that Interferon Regulatory Factor-1 (IRF-1), which is deficient in the brain of individuals with Alzheimer’s disease (AD), negatively regulates the O-GlcNAcylation levels of GluN1 through transcriptional regulation of the human OGA gene. Furthermore, IRF-1 may influence trafficking of NMDARs, thereby affecting dendritic spine density and synaptic plasticity, and ultimately improving the learning and memory of 3xTg-AD mice.

**Conclusion:**

Our results indicate that IRF1 can improve the cognitive function of 3xTg-AD mice by regulating the O-GlcNAcylation of GluN1, offering evidence that IRF-1 could serve as a novel therapeutic target for treating synaptic dysfunction in Alzheimer’s diseases.

**Supplementary Information:**

The online version contains supplementary material available at 10.1186/s13195-025-01857-w.

## Introduction

Alzheimer’s disease (AD) is a progressive neurodegenerative disorder characterized by neuronal damage in the brain, which leads to impaired memory, language, and thinking skills [1]. The primary focus of drug development for AD has been on directly targeting its two key pathological features: the aggregation of beta-amyloid plaques (Aβ) and the formation of tau tangles, also known as tau neurofibrillary tangles (NFTs). Nevertheless, the quest for a definitive cure for this disease continues to be an ongoing challenge.

The synapse, comprising the synaptic cleft formed by inter-neuronal connections, facilitates the transmission of electrical and chemical signals between neurons. Synaptic dysfunction, encompassing synaptic injury, loss, and structural alterations, is considered early pathological events that precede the formation of Aβ and NFTs in AD [2, 3]. Elucidating the underlying mechanisms responsible for synaptic loss and dysfunction in AD and targeting synaptic treatments, particularly within the early treatment window of AD, may pave the way for novel and more efficacious therapeutic strategies to enhance the quality of life for individuals with AD [4, 5]. Synaptic plasticity, which leads to the enhancement or weakening of synaptic connections, refers to the dynamic changes in the quantity, structure, and function of synapses. It exhibits dynamic characteristics and has long been recognized as an essential component of learning and memory [5, 6]. Aberrations in synaptic plasticity, particularly within the hippocampus, are correlated with the cognitive impairment observed in patients with AD [7]. Long-term potentiation (LTP) and long-term depression (LTD), which reflect enhancements or reductions in synaptic transmission efficacy [6], are the primary manifestations of synaptic plasticity. Extensive research has demonstrated that LTP plays a crucial role in the formation of neuronal ensembles that encode memories, while LTD is implicated in their disassembly, leading to memory deactivation [8]. Fine spine membranous protrusions known as dendritic spines exhibit variations in density, size, and shape, which form the basis of synaptic structural plasticity involved in cognition and memory. Impaired synaptic plasticity and intellectual disability have been linked to reduced spine numbers [9].

O-GlcNAcylation, implicated in a variety of cellular stress responses and cellular processes [10], refers to a post-translational O-linked *β*-N-acetylglucosamine (O-GlcNAc) modification in the nucleus and cytoplasm. O-GlcNAcylation typically occurs on substrate-specific serine and threonine residues and is catalyzed by O-GlcNAc transferase (OGT), while the reverse process is generally controlled by O-GlcNAcase (OGA) [11, 12]. O-GlcNAcylation mediates the progression of a variety of chronic diseases including cancer [13], diabetes [14] and neurodegenerative diseases such as AD [15]. Notably, two related enzymes, OGT and OGA, exhibit high expression and activity in the brain compared to other organs [16, 17]. Furthermore, the high expression of brain OGA and OGT is mainly concentrated in the cytosol of synaptosomes, the nerve terminal structures. Therefore, the modulation of O-GlcNAcylation leads to alterations in synaptic and neural function [18, 19]. O-GlcNAcylation is particularly abundant in neuronal synapses [18]. In recent years, an increasing number of methods have been used to identify O-GlcNAcylation and O-GlcNAcylation sites on proteins associated with synapses and memory in AD from human and murine brain tissues [20, 21]. It has been revealed that increased O-GlcNAcylation, induced by a brain-specific knockout of OGA in mice, results in delayed brain differentiation and neurogenesis, as well as aberrant proliferation that accompanies developmental delays [22]. Increased O-GlcNAcylation also causes impairments in hippocampus-associated spatial learning and memory, as well as defects in synaptic plasticity [23]. In addition, an OGA deficiency impairs cognition and synaptic morphology in Drosophila [24].

Excitatory glutamate receptors on postsynaptic membrane of hippocampal pyramidal neurons are mainly AMPA receptors (AMPARs) and NMDA receptors (NMDARs). both types belong to the family of ionotropic glutamate receptors (iGluRs) and coexist within the postsynaptic density (PSD) of the postsynaptic membrane of glutamatergic neurons, where they are successively activated by glutamate released from the synaptic terminals of glutamatergic neuron [25, 26]. It has been reported that GluA2, a subunit of AMPAR, is modified by O-GlcNAc and is associated with long-term depression at hippocampal synapses [27]. However, it remains unclear whether NMDAR can be modified by O-GlcNAc to participate in synaptic plasticity and thereby affect cognitive function.

NMDARs are composed of heterotrimeric subunits that belong to three distinct classes: GluN1, GluN2A-GluN2D, and GluN3A-B [28]. They are widely distributed throughout the central nervous system (CNS) and are involved in essential functions, including synaptic transmission, learning, memory, plasticity, and excitotoxicity [29]. Throughout all developmental stages, the GluN1 gene is ubiquitously expressed in neurons. In the mature hippocampus, GluN2A and GluN2B are predominantly expressed in the pyramidal cells of CA1 and CA3 [30]. The activation of NMDARs leads to the opening of ion channels in the corresponding postsynaptic membrane, initiating a cascade of events that modulate electrophysiological activities in postsynaptic neurons and induce LTP, thereby enhancing learning function [31]. It has been observed that aberrant expression of NMDAR in the hippocampal-cortical region of the brain is associated with memory impairment [32].

Interferon regulatory factor-1 (IRF-1), initially identified as a transcription factor capable of activating interferon-beta expression [33], functions as both a transcriptional activator and repressor for various target genes. It regulates gene expression by binding to interferon-stimulated response elements (ISREs) in their promoters through its N-terminal helix-turn-helix DNA-binding domain [34]. In addition to its function as a transcription factor [35], IRF-1 also regulates cellular pyroptosis [36], cell cycle arrest, apoptosis [37], and the inflammatory response [38]. Learning and memory impairments, as well as diminished motor abilities, are the primary clinical manifestations of AD. The protective effect of IRF-1 on cognitive decline is evident under normal conditions; however, no significant impact on cognition was observed following treatment with chronic cerebral hypoperfusion [39]. Other studies have suggested that angiotensin II type 2 (AT_2_) receptors can enhance spatial memory [40], and IRF-1 induces the expression of AT_2_ receptors [41], implying that IRF-1 exerts beneficial effects on cognitive decline through AT_2_ receptor signaling. Furthermore, abnormal overexpression of IRF-1 has been observed in the dorsolateral prefrontal cortex of individuals with neurocognitive disorders [42].

Our current research findings indicate that the downregulation of IRF-1 in the hippocampus and cortex is associated with AD pathology. IRF-1 transcriptionally promotes the expression of OGA, thereby inhibiting O-GlcNAc modification of GluN1. Furthermore, we use the 3xTg-AD model to demonstrate that IRF-1 enhances the surface expression of GluN1 and increases dendritic stability, which mediates synaptic transmission and ultimately leads to improved cognitive function.

## Materials and methods

### Human brain samples

The research involving frozen human brain tissues adhered to the guidelines of the National Institutes of Health. The frontal lobes from ten Braak stage V and VI Alzheimer’s disease (AD) brains, as well as ten control brains matched for age and postmortem delay, were obtained from the Sun Health Research Institute Donation Program (Sun City, Arizona, USA). These samples were from unidentified donors. All brain tissues were stored at -80 °C until they were used.

### Animals

All animal procedures were conducted in accordance with the guidelines approved by the Nantong University Animal Care and Use Committee. IRF-1 knockout mice were purchased from Gem, while 3xTg-AD mice were obtained from the group’s breeding colony. AAV-IRF-1 or AAV-OGA 3xTg-AD mice were created by injecting IRF-1 or OGA overexpressing adeno-associated viruses into the hippocampal region of 3xTg-AD mice. Hippocampal and cortical tissues were collected at 2–3 months of age and used for the analysis in subsequent experiments.

### Cell culture and transfection

HEK-293T cells were maintained in Dulbecco’s Modified Eagle Medium (DMEM, Corning) supplemented with 10% fetal bovine serum (FBS, Gibco) at 37 °C. Primary cortical neurons were extracted from the hippocampus and cortex of Sprague Dawley rat brains on the 18th day of embryo, digested with 0.25% trypsin containing EDTA, filtered through 200 and 400 mesh sieves, counted, and plated. The Neurobasal medium (Gibco) was supplemented with 2% B27 and penicillin (100 U/ml)-Streptomycin (0.1 mg/ml) (Beyotime). The transfection of siRNA or plasmid into HEK-293T cells was performed using Lipofectamine 2000 (Thermo Fisher Scientific), and the infection of adenovirus (Gene Pharma) into HEK-293T cells and primary cortical neurons was carried out following the manufacturer’s instructions. After transfection for 48–72 h, the cells were utilized for subsequent experimental analysis.

### Golgi staining

The brains of IRF-1 knockout mice and 3xTg-AD mice injected with IRF-1 were rapidly excised post-mortem and immersed in a prepared solution containing potassium dichromate and potassium chromate (A and B), as per the instructions of the FD Rapid GolgiStain™ Kit, for a duration of 14 days. Subsequently, the brains were transferred to a 6% sucrose solution (C) and left to soak for a minimum of 3 days. Following embedding in OCT, the brains were serially sectioned into 100 μm thick slices using a frozen sectioning machine. The brain slices were then mounted onto gelatin-coated slides, allowed to air-dry slightly, and processed through a series of steps including distilled water washing, ethanol dehydration, and xylene transparency. Finally, the slices were sealed, and the images were observed under a microscope and photographed. The dendritic spines were quantified per 10 μm.

### Biochemical measurement of surface-expressed receptors

Brain slices or primary neuronal cells cultured and transfected with IRF-1 adenovirus for 48 h were incubated with artificial cerebrospinal fluid (ACSF) containing 1 mg/ml Sulfo-NHS-LC-Biotin (Thermo Fisher Scientific) for 20 min on ice. Subsequently, the biotin reaction was quenched by rinsing the cells three times with TBS. The cells were then lysed, and the protein concentration was quantified. A total of 15 µg of protein was used to measure the total NMDARs, while the remaining proteins were incubated with 50% Neutravidin Agarose (Thermo Fisher Scientific) at 4 °C for either 2 h or overnight. The precipitate was then mixed with 20–30 µl of 2xSDS sample buffer, boiled, and subjected to western blot analysis. Both total and biotinylated (surface) proteins were analyzed using anti-GluN1 (Cell Signaling Technology), anti-GluN2A (NeuroMab), and anti-GluN2B (Proteintech) antibodies.

### Immunocytochemical measurement of internalized receptors

Primary neuronal cells were infected with shIRF-1 adenovirus for 48 h. The cells were then incubated with medium-diluted rabbit-derived antibodies against GluN1 (Cell Signaling Technology), GluN2A (Cell Signaling Technology), and GluN2B (Proteintech) for 20 min at 37 °C. To strip off the antibodies bound to the remaining surface NMDARs, an acid solution (0.5 M NaCl, 0.2 N acetic acid) was added and the mixture was placed on ice for 3 min. Following this, the cells were washed, fixed, permeabilized, and incubated with murine-derived antibodies against GluN1 (BD Biosciences), GluN2A (NeuroMab), and GluN2B (BD Biosciences) at room temperature for 2 h. Internalized NMDARs were detected using a red-conjugated anti-rabbit secondary antibody, whereas total NMDARs were detected with a green-conjugated anti-mouse secondary antibody.

### Luciferase activity assay

The pGL3-basic vector, pGL3/OGA1488, or truncation mutant plasmid was transfected alone or together with siCtrl/siIRF-1 into HEK-293T cells transfected with pRL-Tk for 48 h. The luciferase activity was then measured and quantified using the Dual Luciferase Reporter Gene Assay Kit (Yeasen Biotech), following the manufacturer’s instructions.

### Mass spectrometry

Brain hippocampal protein homogenates from IRF-1 knockout mice were incubated overnight at 4 °C with pre-conjugated protein G beads (Beyotime) and antibodies (GluN1, Cell Signaling Technology). The immunoprecipitated products were then detached by SDS-PAGE and silver-stained. Gel fragments containing GluN1 were digested using in-gel trypsin. The proteolytic peptides were extracted from the gel, and O-GlcNAc-modified peptides were enriched using TiO2 IMAC. The obtained fraction was concentrated and reconstituted in 10 µl of 5% formic acid for LC-MS/MS analysis.

### Long-term potentiation

Acute brain slices from AAV-IRF-1 3xTg-AD mice and their control mice were transferred to a recording chamber and submerged in artificial cerebrospinal fluid (aCSF). The slices were positioned in a recording chamber with 8 × 8 microelectrode arrays (Parker Technology, Beijing, China) on the bottom plane (each microelectrode array measured 50 × 50 mm, with an interpolar distance of 150 μm) and kept submerged in the aCSF. Field excitatory postsynaptic potentials (fEPSPs) of CA1 neurons were recorded by exciting CA3 neurons. LTP was evoked by three sets of high-frequency stimulation (HFS; 100 Hz, duration 1 s). The magnitude of LTP was quantified as the percentage change in the fEPSP slope (10%−90%) measured during the 60-minute interval after LTP induction.

### Morris water maze test

The Morris Water Maze (MWM) was conducted to assess spatial learning and memory. For the spatial learning task, 3xTg-AD mice injected with AAV-IRF-1, or AAV-OGA, along with their control counterparts, were trained to locate a submerged platform in the water over six consecutive days. Each day, the mice were introduced into the water from four different quadrants, and the average time taken to find the platform was recorded. If a mouse failed to locate the platform within 60 s, it was guided to the platform and allowed to remain there for 20 s. Following the removal of the platform on the seventh day, swimming speed and the number of platform crossings within one minute were analyzed using the ANY-maze Video Tracking System (Stoelting Co., WI, USA).

### Statistical analysis

Data were presented and statistically analyzed using GraphPad Prism. The two-tailed Student’s *t*-test, one-way ANOVA, or two-way ANOVA were used to compare differences, and all data are presented as mean ± S.E.M. *p* ≤ 0.05 was considered statistically significant.

## Results

### Downregulation of IRF-1 is associated with AD pathology

**Fig. 1:**
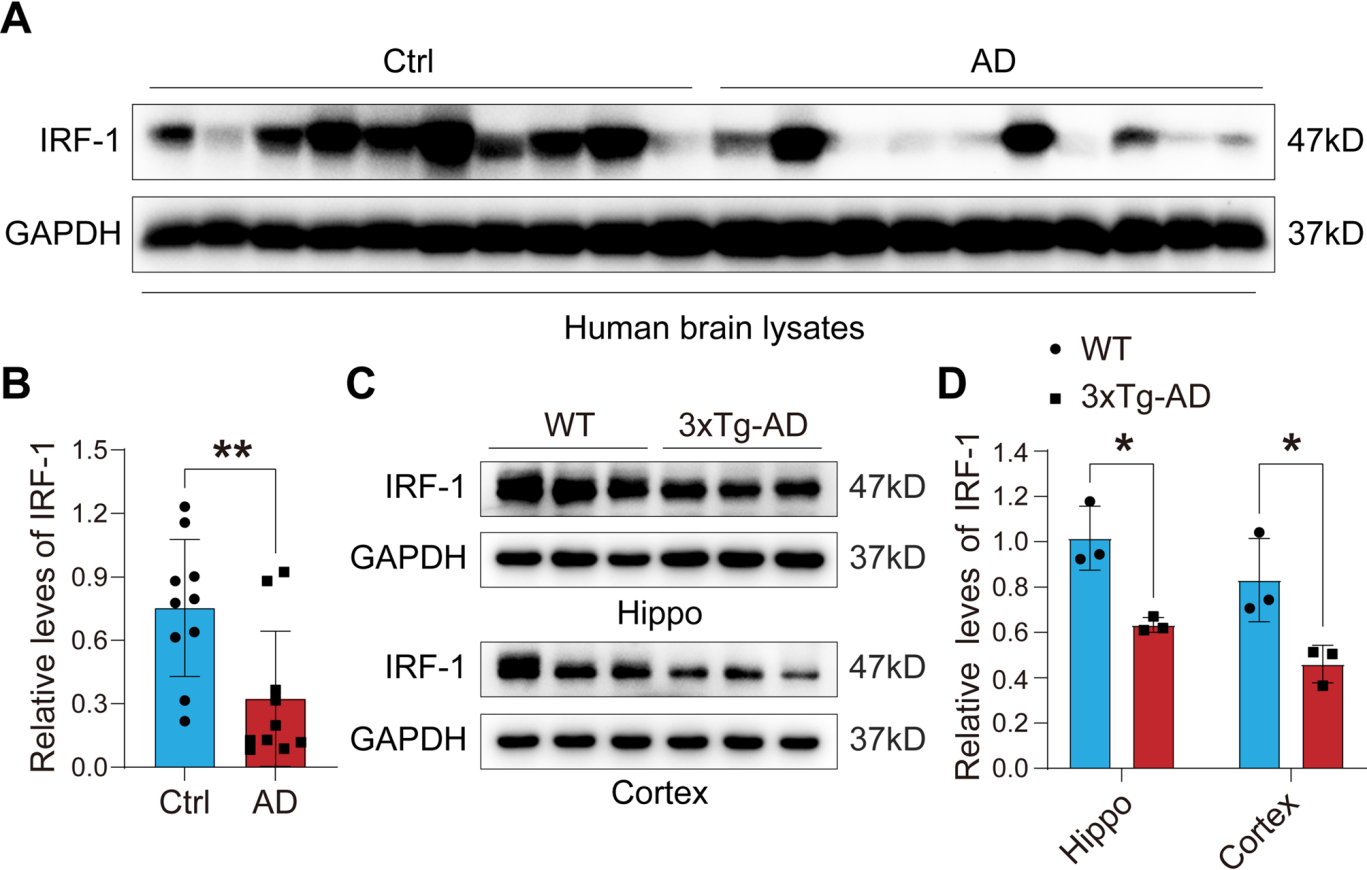
Reduced expression of IRF-1 in AD pathological tissues. (**A-B**) Brain homogenates from 10 AD and 10 control brains were subjected to western blots using IRF-1 antibody. Relative protein levels of IRF-1 were quantified after normalization with GAPDH (*n* = 10). (**C-D**) IRF-1 expression in hippocampal and cortical tissues of 3xTg-AD mice was assessed by western blots and quantification (*n* = 3). All the data are presented as mean ± S.E.M. *p < 0.05; **p < 0.01 vs. Ctrl or WT, two-tailed student’s *t* test or two-way ANOVA with Šídák’s multiple comparisons test

To examine the expression level of IRF-1 in AD human brains, we compared frontal lobe samples with a short post-mortem delay (< 3 h) from ten Braak stage V and VI AD brains to ten age-matched control brains (Table S1) using western blot assay. We found that the protein level of IRF-1 was significantly decreased in the AD human brain (Fig. 1A-B). We also determined the expression and distribution of IRF-1 in the hippocampus and cortex of 3xTg-AD model mice carrying three human AD-related genes: Psen1, APPSwe, and tauP301L [43]. We found that IRF-1 expression was inhibited in the hippocampus and cortex of the 3xTg-AD model mice compared to WT mice (Fig. 1C-D). Meanwhile, immunofluorescent staining showed that IRF-1 was widely distributed in the hippocampus, but a reduced distribution of IRF-1 was found in the hippocampus of 3xTg-AD mice compared to that of their littermate control mice (Fig. S1A-B). These results suggest that the downregulation of IRF-1 might be related to AD pathology.

### IRF-1 is negatively correlated with protein O-GlcNAcylation

**Fig. 2:**
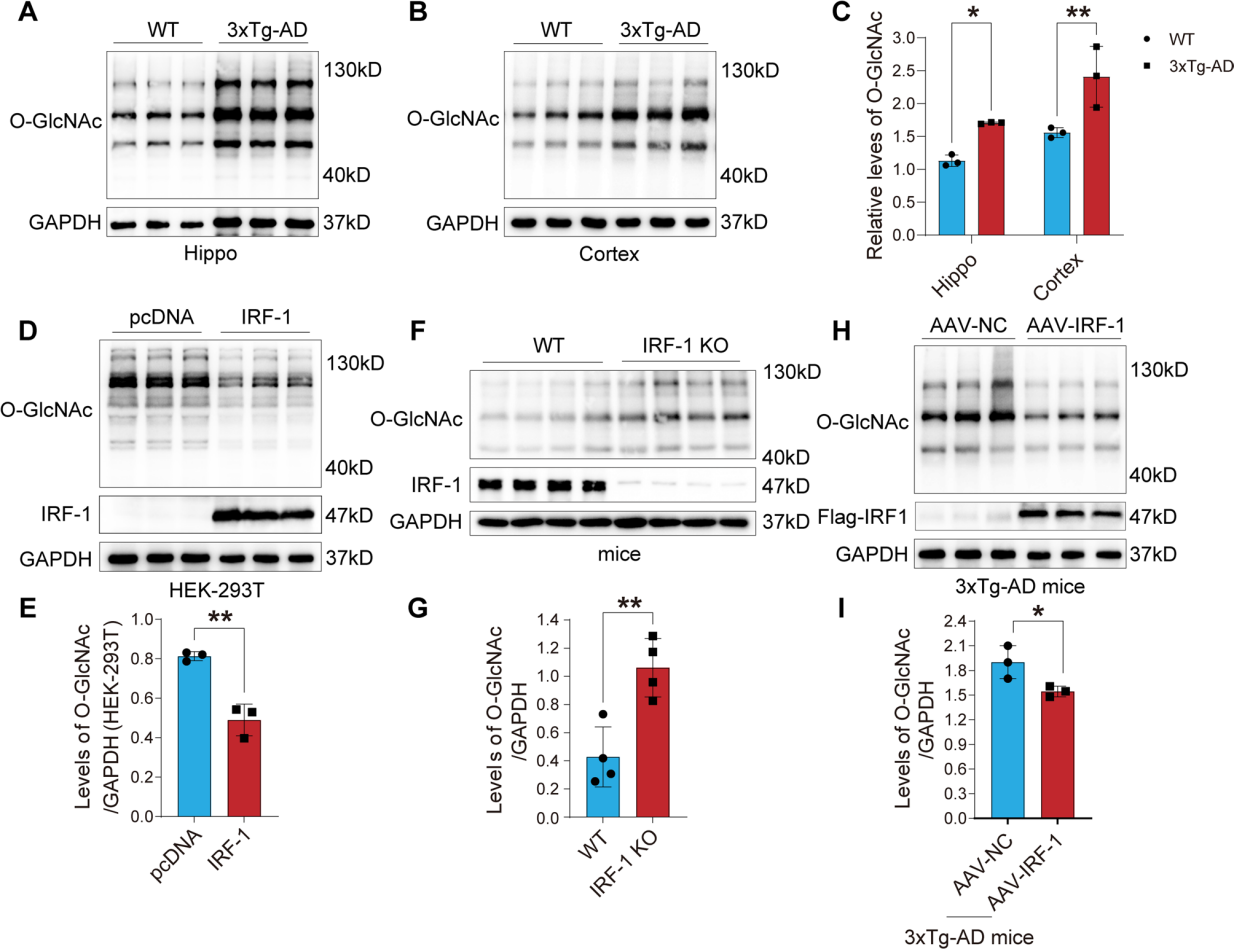
IRF-1 suppresses protein O-GlcNAcylation. (**A-C**) O-GlcNAcylation levels in hippocampal and cortical tissues of 3xTg-AD mice were analyzed using western blots and quantification (*n* = 3). (**D-E**) HEK-293T cells were transfected with pcDNA/Flag-IRF-1. Protein levels of O-GlcNAcylation, IRF-1, and GAPDH were examined using western blots with anti-RL2, anti-IRF-1, and anti-GAPDH antibodies (*n* = 3). (**F-G**) Brain homogenates from IRF-1 knockout (KO) mice or their wild type (WT) littermates were collected, and differences in O-GlcNAcylation levels were detected and quantified (*n* = 4). (**H-I**) Brain homogenates from 3xTg-AD mice injected with AAV-IRF-1 were collected and variations in O-GlcNAcylation levels were assessed and quantified (*n* = 3). All data are presented as mean ± S.E.M. *p < 0.05; **p < 0.01 vs. WT, pcDNA or AAV-NC, two-tailed student’s *t* test or two-way ANOVA with Šídák’s multiple comparisons test

**Fig. 3:**
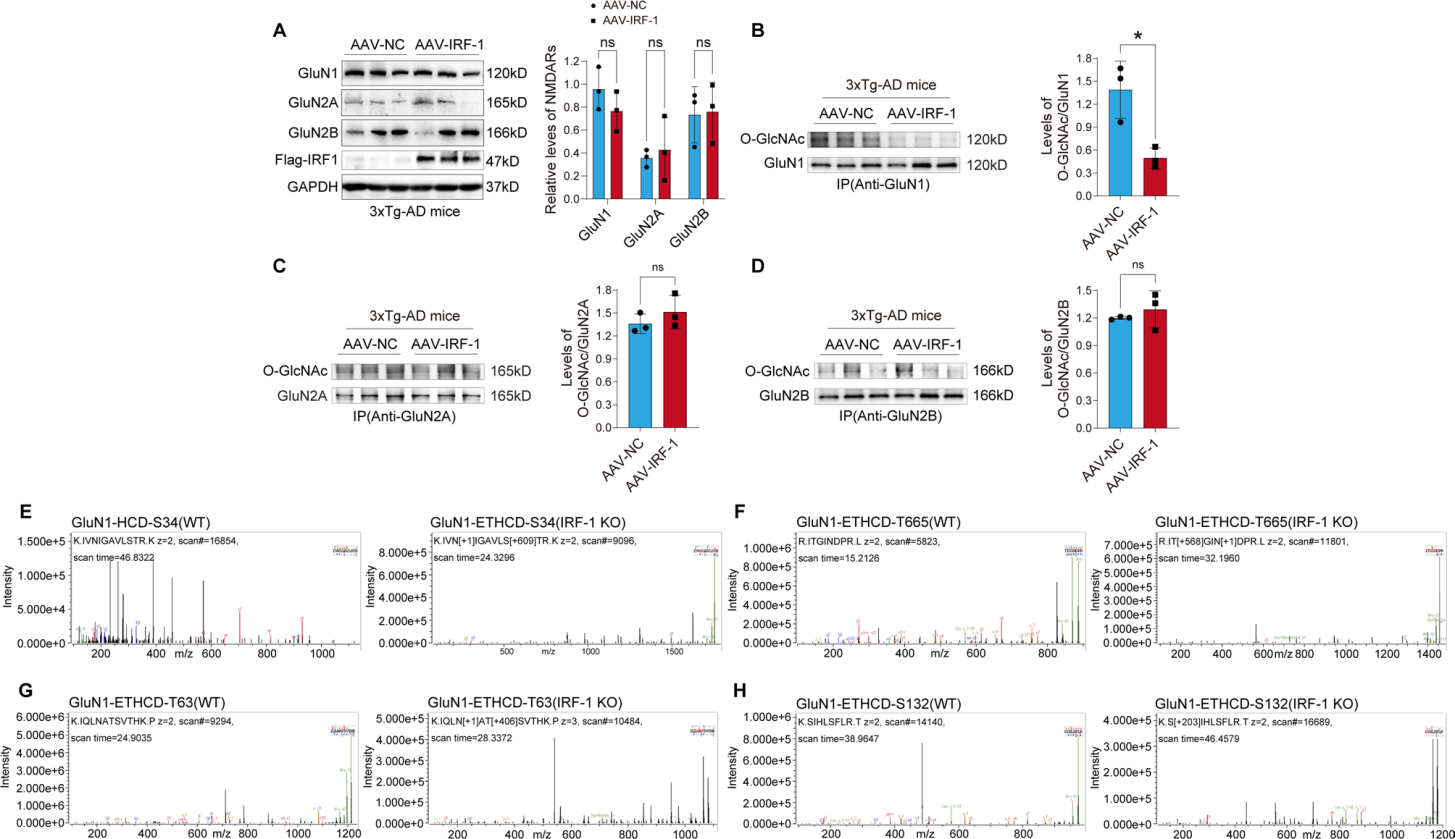
IRF-1 modulates the O-GlcNAcylation of GluN1. (**A**) Cortical brain homogenates were collected from 3xTg-AD mice injected with AAV-IRF-1 in the hippocampus to assess NMDARs expression through western blotting analysis (*n* = 3). (**B-D**) The O-GlcNAcylation levels of immunoprecipitated GluN1 (B), GluN2A (C) or GluN2B (D) from brain homogenates of 3xTg-AD mice injected with AAV-IRF-1 were analyzed using western blots with anti-RL2, anti-GluN1, anti-GluN2A or anti-GluN2B antibody. The relative O-GlcNAcylation levels (RL2) of immunoprecipitated GluN1, GluN2A or GluN2B were quantified after normalization with GluN1, GluN2A and GluN2B (*n* = 3). (**E-H**) Mass Spectrometry analysis was performed on GluN1 immunoprecipitated from brain homogenates of IRF-1 knockout and their littermate control mice. All data are presented as mean ± S.E.M. *p < 0.05 vs. pcDNA, AAV-NC or WT, two-tailed student’s *t* test or two-way ANOVA with Šídák’s multiple comparisons test

O-GlcNAcylation and synaptic function have been a concern of our group [44]. We first observed a higher level of O-GlcNAcylation in hippocampal and cortical homogenates from 3xTg-AD mice compared to control cases using western blot (Fig. 2A-C). To further determine the relationship between IRF-1 and O-GlcNAcylation, we detected O-GlcNAcylation levels in HEK-293T cell lysate overexpressing IRF-1 (Fig. 2D-E), as well as in homogenates from the brain of IRF-1 knockout mice (Fig. 2F-G) and 3xTg-AD mice injected with AAV-IRF-1 (Fig. 2H-I). Western blot analysis revealed that protein O-GlcNAcylation level was significantly decreased when IRF-1 was overexpressed in HEK-293T cells (Fig. 2D-E) or in the brains of 3xTg-AD mice injected with AAV-IRF-1 (Fig. 2H-I). Conversely, protein O-GlcNAcylation levels increased when IRF-1 was knocked down in the brains of IRF-1 KO mice (Fig. 2F-G). These results indicate that IRF-1 negatively regulates protein O-GlcNAcylation.

### IRF-1 suppresses O-GlcNAcylation of GluN1, but not GluN2A or GluN2B

O-GlcNAcylation is particularly abundant in neuronal synapses [18]. To clarify whether IRF-1 regulates O-GlcNAcylation of NMDARs, IRF-1 was overexpressed in 3xTg-AD brains, or IRF-1 was knocked down in IRF-1 KO mice brains, then western blot was performed to examine the protein levels of IRF-1, GluN1, GluN2A, or GluN2B respectively. We found that IRF-1 did not affect the protein levels of GluN1, GluN2A, or GluN2B, which are subunits of NMDAR (Fig. 3A; Fig. S2A). Subsequently, GluN1, GluN2A, or GluN2B was immunoprecipitated from cortical brain homogenates of IRF-1 knockout mice and 3xTg-AD mice injected with AAV-IRF-1 respectively. Affinity purified proteins were analyzed by western blot developed with RL2. We found that IRF-1 negatively regulated the O-GlcNAcylation level of GluN1 (Fig. 3B; Fig. S2B; Fig. S3), but not that of GluN2A (Fig. 3C; Fig. S2C) or GluN2B (Fig. 3D; Fig. S2D). To establish a direct connection among IRF-1, OGA, and the O-GlcNAcylation of GluN1, we overexpressed GluN1 alone or in conjunction with IRF-1, and subsequently inhibited OGA using varying concentrations of Thiamet G in HEK-293T cells. Following this, immunoprecipitation was conducted with an antibody specific to GluN1, and the O-GlcNAcylation of GluN1 was assessed via western blot. We observed that IRF-1 decreased the O-GlcNAcylation level of GluN1 (Fig. S4), and that OGA inhibition could counteract the effect of IRF-1 on the O-GlcNAcylation of GluN1 (Fig. S4), further suggesting that IRF-1 suppresses the O-GlcNAcylation of GluN1 by way of OGA.

To examine the O-GlcNAcylation sites of GluN1 regulated by IRF-1, the immunoprecipitated GluN1 from the cortical homogenates of IRF-1 knockout mice and control cases was separated by SDS-PAGE, and then subjected to in-gel trypsin digestion and LC-MS/MS. It was revealed that IRF-1 decreased the O-GlcNAcylation level of GluN1 at S34, T665, T63, and S132 sites (Fig. 3E-H). These results show that GluN1, GluN2A, and GluN2B are all modified by O-GlcNAc, and that IRF-1 negatively modulates the O-GlcNAcylation level of GluN1 at S34, T665, T63, and S132 sites.

### IRF-1 modulates the surface expression and internalization of NMDA receptors, thereby influencing the density of dendritic spines in neurons

**Fig. 4:**
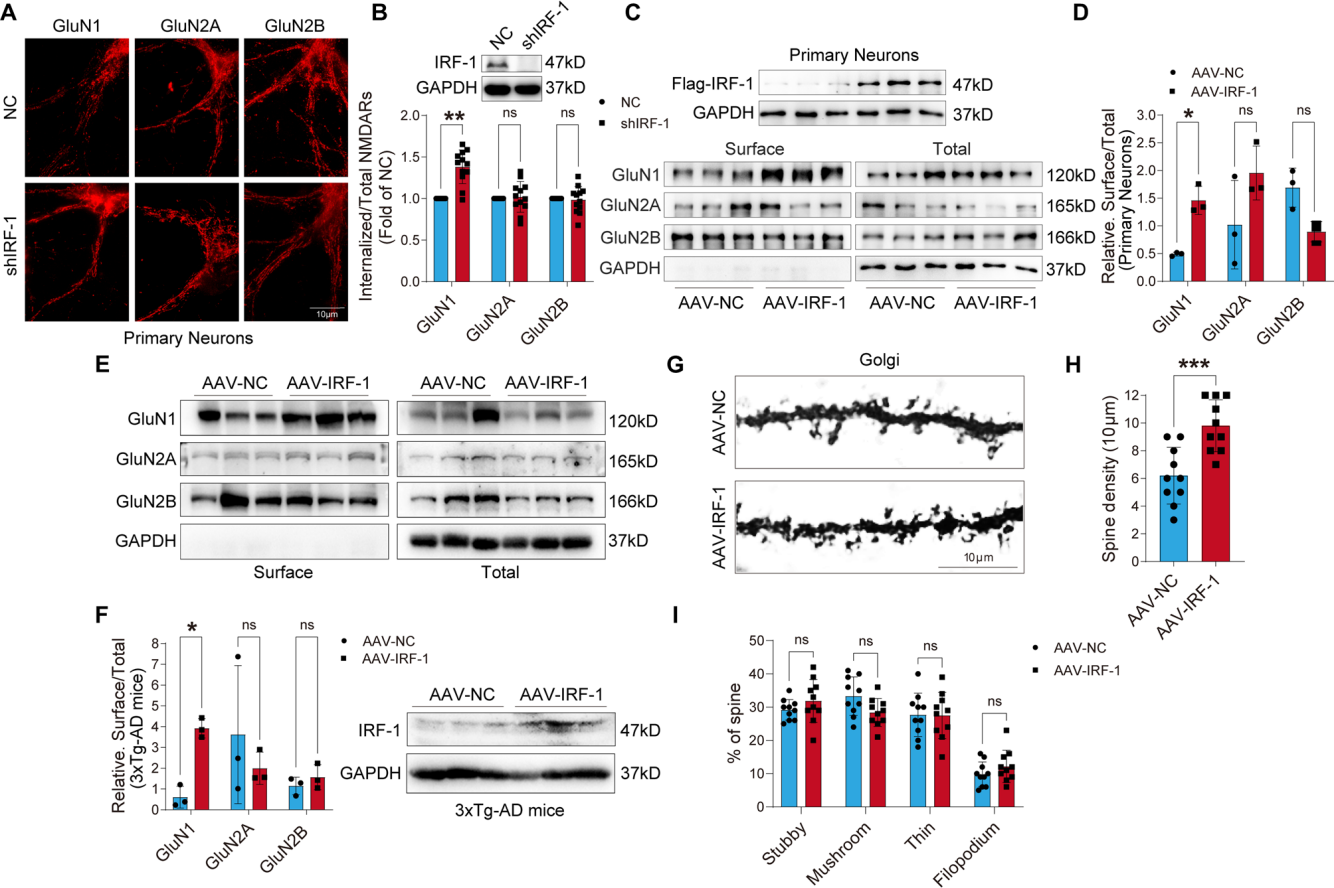
IRF-1 reduces the internalization of GluN1 and increases its surface expression. (**A-B**) Immunocytochemical images (A) and quantitative analysis (B) of internalized and total GluN1, GluN2A, and GluN2B in primary neuronal cells infected with NC and shIRF-1 adenovirus (*n* = 12). (**C-F**) Immunoblots displaying surface and total levels of NMDAR subunits in primary neuronal cells infected with IRF-1 adenovirus (C-D) and in cortical slices of 3xTg-AD mice injected with AAV-IRF-1 in the hippocampus (E-F) (*n* = 3). (**G-I**) Representative images of dendritic spines in hippocampal pyramidal neurons and quantification of dendritic spines. Dendritic spine density per 10 μm in hippocampal pyramidal neurons of 3xTg-AD mice injected with AAV-IRF-1 and AAV-NC mice, determined by Golgi staining and percentages of stubby, mushroom, thin, or filopodium spines were quantified (*n* = 10). All data are presented as mean ± S.E.M. *p < 0.05; **p < 0.01; ***p < 0.001 vs. NC, AAV-NC, two-tailed student’s *t* test or two-way ANOVA with Šídák’s multiple comparisons test

To explore the impact of IRF-1 on the internalization of NMDA receptors in cultured cortical neurons, immunocytochemical assays were performed. Following the infection of primary neuronal cells with shIRF-1 adenovirus for 48 h, surface NMDARs were initially labeled with antibodies targeting the extracellular domain. Then, the surface-bound antibodies were stripped away, allowing for the visualization of only the internalized NMDARs. As shown in Fig. 4A-B, knock down of IRF-1 led to a significant increase in the internalization of GluN1, whereas the internalization of GluN2A and GluN2B remained unaffected.

We also performed surface biotinylation to assess levels of GluN1, GluN2A, and GluN2B on the membrane surface of cortical neurons. Primary cortical neuronal cells were infected with IRF-1 adenovirus, and surface proteins were labeled with Sulfo-NHS-LC-Biotin, followed by Neutravidin agarose reaction to separate biotinylated surface proteins from unlabeled intracellular proteins. Then the surface and the total proteins were subjected to electrophoresis and detected by anti-GluN1, anti-GluN2A or anti-GluN2B antibody. As shown in Fig. 4C-D, overexpression of IRF-1 increased surface expression of GluN1 on synaptic membrane, but not that of GluN2A and GluN2B, consistent with the results shown in Fig. 4A-B. To further confirm that IRF-1 promotes the surface expression of GluN1, 3xTg-AD mice were injected hippocampally with AAV-IRF-1 and the surface and total proteins of the subunits of NMDARs in cortical sections were examined by western blot using specific antibody to each subunit, respectively. We found that IRF-1 promoted the surface expression of GluN1 but not that of GluN2A or GluN2B in the brain of 3xTg-AD mice (Fig. 4E-F).

Dendritic homeostasis is a pivotal indicator of synaptic function [9]. Given that our data indicate that IRF-1 regulates the O-GlcNAcylation level of GluN1 and also influences the surface expression of GluN1 on the neuronal membrane, we investigate the dendritic spine density in hippocampal pyramidal neurons of 3xTg-AD mice injected with AAV-IRF-1 and IRF-1 knockout mice by Golgi staining. As shown in Fig. 4G-H and Fig. S5A-B, IRF-1 was found to enhance dendritic spine density. However, there was no significant difference in the primary morphological subtypes (stubby, mushroom, thin, or filopodium) (Fig. 4I; Fig. S5C). In summary, these results suggest that IRF-1 regulates the trafficking of GluN1 and therefore changes the density of dendritic spine.

### The transcription of OGA is enhanced by IRF-1 through its binding to the promoter region at -332/-324 of OGA

**Fig. 5:**
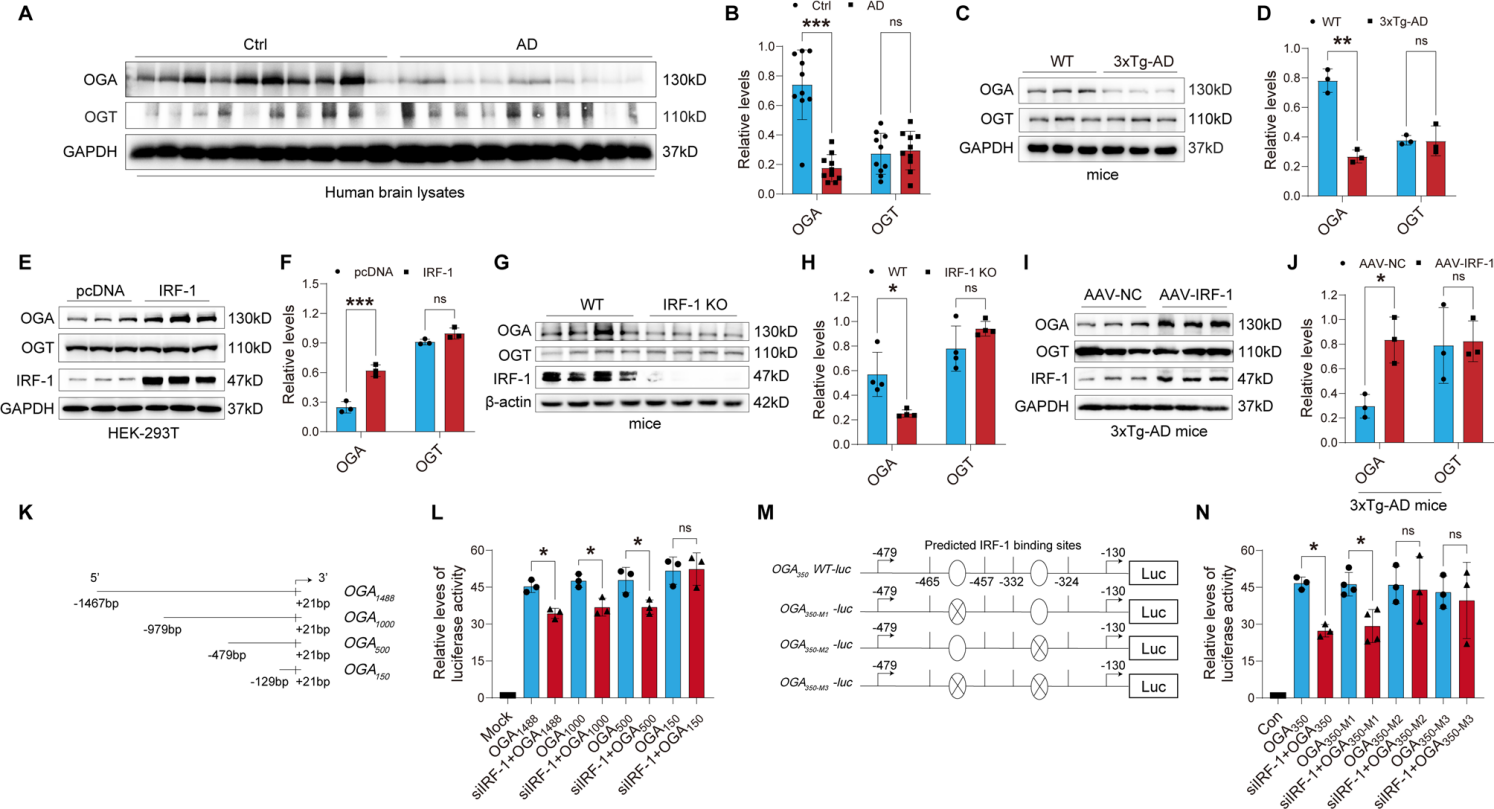
IRF1 acts on the − 332/-324 region of the OGA promoter to transcriptionally promote the expression of OGA. (**A-B**) Brain homogenates from 10 AD and 10 control brains were subjected to western blots using OGA or OGT antibody. The relative protein levels of OGA and OGT were quantified after normalization with GAPDH (*n* = 10). (**C-D**) OGA and OGT protein expression in brain tissues of 3xTg-AD mice was determined by western blotting and quantitative analysis (*n* = 3). (**E-J**) Western blotting confirmed overexpression of IRF-1 in HEK-293T cells (E-F), depletion of IRF-1 in mice brain tissues (G-H) or 3xTg-AD mice injected with AAV-IRF-1 (I-J) for OGA and OGT expression (*n* = 3–4). (**K-L**) Schematic diagram of the constructed plasmid of pGL3/OGA_1488_, pGL3/OGA_1000_, pGL3/OGA_500_, or pGL3/OGA_150_ (K). The pGL3-basic vector, pGL3/OGA_1488_, or truncation mutant plasmid pGL3/OGA_1000_, pGL3/OGA_500_ or pGL3/OGA_150_ was transfected alone or together with siCtrl/siIRF-1 into HEK-293T cells transfected with pRL-Tk. After 48 h transfection, the luciferase activity was measured and normalized with Renilla luciferase (pRL-Tk) (*n* = 3) (L). (**M-N**) The left panel represents the schematic diagram of the constructed plasmid of pGL3/OGA_350_ and the binding sites of IRF-1 located inside the promotor region of OGA, with mutation depicted by an “X” (M). pGL3/OGA_350_, pGL3/OGA_350 − M1_, pGL3/OGA_350 − M2_, or pGL3/OGA_350 − M3_ was transfected alone or together with siCtrl/siIRF-1 into HEK-293T cells transfected with pRL-Tk, the luciferase activity was measured and normalized with Renilla luciferase(pRL-Tk) after 48 h transfection (*n* = 3–4) (N). All data are presented as mean ± S.E.M. *p < 0.05; **p < 0.01; ***p < 0.001 vs. Ctrl, WT, pcDNA, AAV-NC, OGA_1488_, OGA_1000_, OGA_500_, OGA_350_, or OGA_350 − M1_, two-tailed student’s *t* test, one-way ANOVA Šídák’s multiple comparisons test or two-way ANOVA with Šídák’s multiple comparisons test

O-GlcNAcylation levels are dynamically and synergistically modulated by both OGA and OGT [12]. We wonder whether the regulation of O-GlcNAcylation by IRF-1 is due to its effect on OGA, OGT, or both. Given the decreased expression of IRF-1 in the brains of AD patients and 3xTg-AD mice (Fig. 1A-D), we first examined the protein levels of OGA and OGT from frontal lobe samples of AD human brains and brain homogenates of 3xTg-AD mice. We found that the protein level of OGA, but not OGT, was reduced in the brains of AD patients and 3xTg-AD mice (Fig. 5A-D), and that IRF-1 positively correlated well with the protein level of OGA (Fig. S6A-D). To clarify the expression regulatory role of IRF-1 on OGA and OGT, the expression of OGA and OGT was examined respectively in cells overexpressing IRF-1, brain homogenates of IRF-1 KO mice and 3xTg-AD mice hippocampally injected with AAV-IRF-1. We found that IRF-1 promoted protein expression of OGA but not that of OGT (Fig. 5E-J). To further verify whether IRF-1 transcriptionally regulates OGA expression, the mRNA level was detected in IRF-1 overexpressed HEK-293T cells by Real-time PCR. IRF-1 obviously increased the mRNA level of OGA but not OGT (Fig. S6E).

To further explore the regulatory impact of IRF-1 on OGA at the transcriptional level, we inserted the promoter region of human OGA_1488_ (-1467 bp ~ + 21 bp) into the pGL3-basic plasmid to generate the reporter plasmid pGL3/OGA_1488_. We found that pGL3/OGA_1488_ significantly increased luciferase activity (Fig. 5L) compared to pGL3-basic, indicating that the human OGA promoter drives luciferase expression. To ascertain the role of IRF-1 in OGA transcriptional regulation and to identify the key regions of the human OGA promoter involved in transcriptional regulation by IRF-1, we constructed the deletion mutant plasmids pGL3/OGA_1000_, pGL3/OGA_500_, and pGL3/OGA_150_ (Fig. 5K) and transfected them individually or in combination with siIRF-1. As depicted in Fig. 5L, IRF-1 knockdown decreased luciferase activity in cells co-transfected with pGL3/OGA_1488_, pGL3/OGA_1000_, or pGL3/OGA_500_, but had no effect on pGL3/OGA_150_, suggesting that the region from − 479 to -130 of the OGA promoter is a target for IRF-1 transcriptional regulation.

To detail the IRF-1 regulatory element located between − 479 and − 130 in the OGA promotor, we constructed deletion mutant plasmids named pGL3/OGA_350_, pGL3/OGA_350 − M1_, pGL3/OGA_350 − M2_, or pGL3/OGA_350 − M3_, respectively (Fig. 5M). We observed that IRF-1 knockdown reduced luciferase activity in cells co-transfected with pGL3/OGA_350_ and pGL3/OGA_350 − M1_, but not with pGL3/OGA_350 − M2_ or pGL3/OGA_350 − M3_ (Fig. 5N). This suggests that the region from − 332 to -324 of the human OGA promoter is the target binding sites of IRF-1 (Fig. 5M-N). To further validate the binding of IRF-1 to the region from − 332 ~ to -324 on the OGA promoter, chromatin immunoprecipitation assays were conducted. HEK-293T cells were treated with shIRF-1 adenovirus for 48–72 h, and chromatin was immunoprecipitated using a ChIP Assay Kit with an antibody against IRF-1. The quantification and normalization of IRF-1 binding to the OGA promoter region were performed using real-time PCR. As shown in Figure S4F, IRF-1 knockdown resulted in a 20% drop in IRF-1 binding to the OGA promoter region at -332/-324. These findings indicate that IRF-1 may bind to the − 332 to -324 region of the OGA promoter to promote the transcriptional expression of OGA.

### IRF-1 enhances synaptic plasticity and ameliorates learning and memory in 3xTg-AD mice

**Fig. 6:**
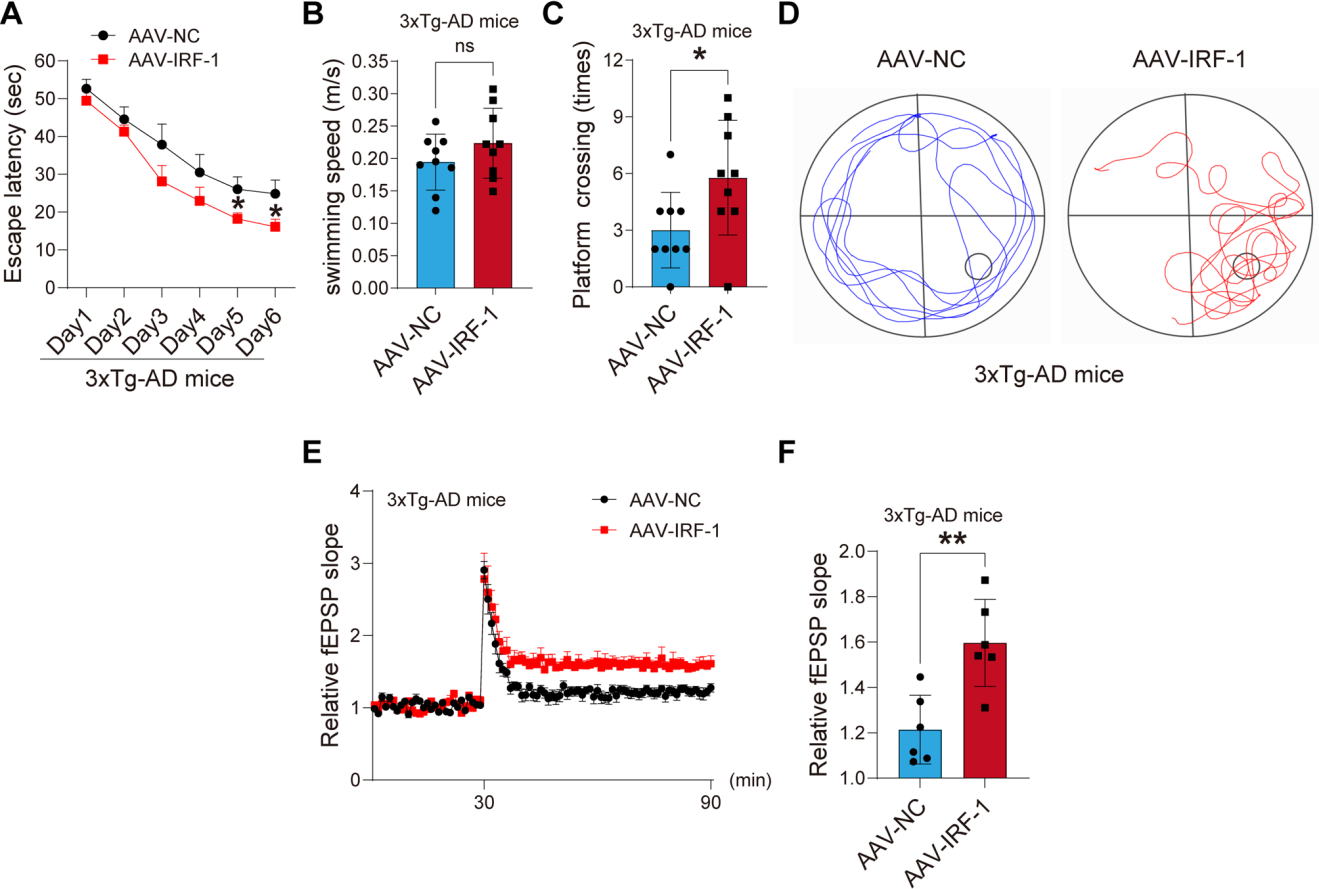
IRF-1 improves synaptic and cognitive function in 3xTg-AD mice. (**A**) 3xTg-AD mice injected with AAV-IRF-1 were trained for 6 consecutive days to find platforms hidden in water, with escape latency recorded and analyzed (*n* = 9). (**B-C**) Swimming speed (m/s) and the number of times the mice crossed the platform in 1 min were recorded after the platform was removed on the 7th day (*n* = 9). (**D**) Representative graphs depicting the swimming trajectories of mice during the testing phase. (**E-F**) Normalized CA3-CA1 fEPSP mean slope recorded from the CA1 dendritic region in hippocampal slices, with quantitative analysis of the relative fEPSP slope presented below (*n* = 6). All data are presented as mean ± S.E.M. *p < 0.05; **p < 0.01 vs. AAV-NC, two-tailed student’s *t* test or two-way ANOVA with Bonferroni’s post hoc test

To investigate whether IRF-1 can mitigate cognitive deficits, we overexpressed IRF-1 in the hippocampal region of the brain of 3xTg-AD model mice via hippocampal injection of AAV-IRF-1. We then conducted the Morris Water Maze test using age-matched littermates injected with vector virus as controls. The performance of animals during training was analyzed based on the latency to reach the submerged platform. On the premise of the same swimming speed, the latency for both AAV-IRF-1 injection mice and control mice decreased over the days, indicating that both groups possessed learning ability. However, AAV-IRF-1 injected mice took less time to reach the platform during the training phase compared to control mice over the 6 consecutive training days, demonstrating that AAV-IRF-1 injection enhanced the spatial learning ability of the mice (Fig. 6A). By recording swimming speed and the number of crossing times within 1 min after the platform was removed on day 7, we found that the 3xTg-AD mice injected with AAV-IRF-1 significantly exhibited more crossing times in the target quadrant compared to control group (Fig. 6C-D), maintaining the same swimming speed (Fig. 6B).

These data collectively provide strong evidence that IRF-1 improves learning and memory in 3xTg-AD mice.

Abnormal synaptic plasticity in the brain, particularly within the hippocampus, is involved in the cognitive impairments observed in AD patients [7]. Given that IRF-1 can enhance learning and memory, we recorded hippocampal CA3-CA1 LTP using the MED64 system to explore whether synaptic plasticity of the CA3-CA1 Schaffer collateral is enhanced by IRF-1. We found that mice injected with AAV-IRF-1 showed an increased slope of field excitatory postsynaptic potentials (fEPSP) after high-frequency stimulation (HFS) compared to controls (Fig. 6E-F). These results indicate that IRF-1 plays an important role in synaptic plasticity.

To strengthen the link between IRF-1 and OGA, we explored whether the overexpression of OGA in 3xTg-AD mice could suppress GluN1 O-GlcNAcylation and improve cognitive function, irrespective of IRF-1. Western blot analysis revealed that the protein levels of OGA were increased while the overall O-GlcNAcylation levels were decreased (Fig. S7A-B) following the injection of AAV-OGA. GluN1 was immunoprecipitated from the brain homogenates of these mice, and its O-GlcNAcylation levels were assessed using RL2. We found that the O-GlcNAcylation of GluN1 was obviously reduced in conjunction with the overexpression of OGA (Fig. S7C-F). The Morris Water Maze testing showed that AAV-OGA-injected mice crossed the platform in the target quadrant more frequently than control mice (Fig. S7G-J), reflecting the improved spatial memory.

These data remind us that IRF1 negatively regulates the O-GlcNAcylation level of GluN1 and enhances cognitive function in 3xTg-AD mice by increasing OGA expression.

## Discussion

Synaptic plasticity, which represents the efficacy of synaptic transmission between neurons, is crucial for learning and memory [45]. NMDARs, the major type of ionotropic glutamate receptors in the central nervous system, play unique and important roles in synaptic transmission and plasticity [46–49]. NMDARs have been associated with synaptic degeneration in Alzheimer’s Disease [50]. In this study, we explore the relevance between the O-GlcNAc modification of NMDARs and synaptic function, and subsequently the effect on learning and memory in 3xTg-AD model mice.

Posttranslational modifications of glutamate receptors have emerged as pivotal modulators of synaptic transmission and plasticity [51]. Evidence indicates that post-translational modulation of NMDA receptors, such as phosphorylation, palmitoylation, and ubiquitination, control the stability, trafficking, and synaptic expression in the central nervous system [52]. Many neuronal proteins, which are O-GlcNAcylated, have been identified by large-scale proteomics [53–56]. Especially, 19% of synaptic proteins are O-GlcNAcylated [55], including bassoon, piccolo, shank2, synapsin I, synaptopodin, and the GluA2 subunit of AMPAR [18, 53–57]. Whether NMDAR is modified by glycosylation has not been reported so far. We have identified for the first time in this study that the GluN1, GluN2A, and GluN2B subunits of NMDAR are modified by O-GlcNAc (Fig. 3B-D; Fig. S2B-D).

IRF-1, originally identified as one of the transcriptional activators responsible for the expression of interferon and interferon-inducible genes, controls the expression of genes related to inflammation and injury [33, 58]. It has been reported that IRF-1 immunoreactivity is present in neutrophils and neurons in the postischemic brains of both rodents and humans [59]. Research on IRF-1 in the brain has mainly focused on neuroinflammation, such as encephalomyelitis [60–63]. It’s the first time for us to reveal that the protein level of IRF-1 decreases both in the brains of individuals with AD (Fig. 1A-B) and in the brains of 3xTg-AD mice (Fig. 1C-D). We have also found that the global O-GlcNAcylation level is inversely correlated with IRF-1 protein expression in both the HEK-293T cells (Fig. 2D-E) and the hippocampus and cortex of mice brains (Fig. 2A-C, F-I).

O-GlcNAcylation of the GluA2 subunit of AMPAR regulates the trafficking of AMPA receptor [57]. An inhibitor of OGA induces a novel form of LTD based on GluA2 endocytosis [27, 64], whereas an inhibitor of OGT increases GluA2 surface expression [57]. In the present study, we observed that IRF-1 decreased the O-GlcNAcylation level of GluN1 and enhanced surface GluN1 expression while inhibiting GluN1 endocytosis (Fig. 4). Alterations in spine density and hippocampal CA3-CA1 LTP are recorded when the expression level of IRF-1 changes (Figs. 4G-H and 6E-F; Fig. S5A-B).

Although the precise O-GlcNAcylation site on GluN1 has not been fully identified, our data indicate that the O-GlcNAcylation level of GluN1 elevates at S34, T665, T63, and S132 in the brains of IRF-1 knock out mice (Fig. 3E-H). It is well establish that the regulation of O-GlcNAcylation is solely mediated by OGT and OGA, and that these two enzymes are distributed throughout the body, including the nervous system [65]. We have uncovered that IRF-1 positively regulates the expression level of OGA, but not OGT, in cells, mice and AD human brains (Fig. 5A-J). IRF-1 recognizes DNA elements similar to interferon-stimulated response elements (ISREs) (A/GNGAAANNGAAACT) [66]. In addition to ISREs, IRF-1 also binds to regions containing 5′-GAAA-3′-containing, such as the positive regulatory domain I (PRD1) in the IFN-β gene promoter [67, 68]. Our experiments suggest that IRF-1 majorly binds to the − 332~-324 region of the human OGA promoter (Fig. 5K-N; Fig. S6F). The DNA sequence of this region is 5′-TTTCCTATC-3′, in which 5′-TTTC-3′ is homologous complementary to 5′-GAAA-3′.

The O-GlcNAc modification has been reported to play multiple, complex roles in in regulating cognitive function and the development of AD. The apparent discrepancy is ongoing with reports presenting both increased and decreased O-GlcNAcylation levels in the AD brain [69–76]. Paradoxical evidence also suggests that O-GlcNAcylation has both neuroprotective and neurodegenerative effects [69, 77]. The controversy of O-GlcNAcylation in AD may be due to different animal models, variant brain regions, or types of *O*-GlcNAc regulatory elements. Here, our findings reveal a deficiency of IRF-1 enhances O-GlcNAcylation of GluN1, leading to cognitive defects.

NMDARs play a crucial role in providing the molecular substrate for important cognitive functions by mediating synaptic transmission and plasticity [78, 79]. Since OGA is not specific to GluN1, we overexpressed OGA by injecting AAV-OGA into the hippocampus of 3xTg-AD mice and detected the O-GlcNAcylation level of GluN1. We found that accompanied by the high expression of OGA, the O-GlcNAcylation level of GluN1 was reduced in the brain homogenates of 3xTg-AD mice (Fig. S7F). These data indicate that OGA negatively correlates with the O-GlcNAcylation level of GluN1. Therefore, it is entirely possible that OGA regulates the O-GlcNAcylation level of GluN1 to control the activity of NMDARs, playing roles in cognitive function.

**Fig. 7:**
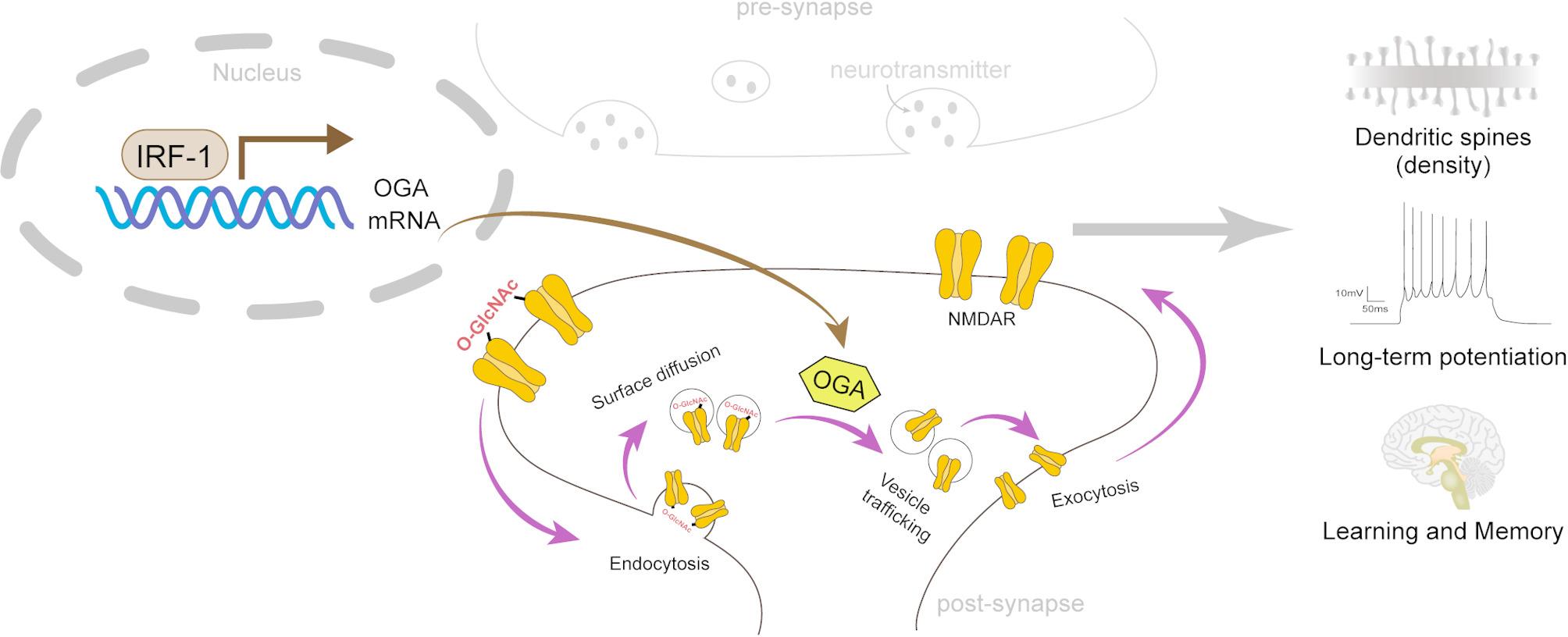
The proposed mechanism by which IRF-1 regulates early synaptic pathology. IRF-1 inhibits the O-GlcNAcylation of NMDAR by promoting the OGA expression, which increases the surface/internalization ratio of GluN1 at the postsynaptic membrane. This ultimately enhances dendritic stability, mediates synaptic transmission, and improves learning and memory in the hippocampus

Taken together, the data of this research indicate that IRF-1 promotes OGA expression by binding to the OGA promoter at the − 332~-324 region, which has the DNA sequence 5′-TTTCCTATC-3′. We have observed that the O-GlcNAcylation level of the GluN1 subunit, but not GluN2B, increased at S34, T665, T63, and S132 in the brains of IRF-1 KO mice. Knock down of IRF-1 leads to an increased percentage of internalized GluN1 in primary neuronal cells (Fig. 4A-B), while over-expression of IRF-1 results in a more pronounced surface expression of GluN1, but not GluN2A, or GluN2B in cells (Fig. 4C-D), and in the brains of 3xTg-AD mice (Fig. 4E-F). In addition, 3xTg-AD mice brains injected with AAV-IRF-1 exhibit a significant increase in spine density (Fig. 4G-H). An enhancement in the magnitude of LTP, and consequently improved cognition, is observed in 3xTg-AD mice injected AAV-IRF-1 (Fig. 6A-D, E-F). Our findings support the notion that IRF-1 regulates the O-GlcNAcylation of NMDAR subunits through transcriptional modulation, influencing the trafficking of NMDARs, which results in increased synaptic plasticity and improved learning and memory (Fig. 7).

## Supplementary Information

Below is the link to the electronic supplementary material.

Supplementary Material 1

## Data Availability

No datasets were generated or analysed during the current study.
