# Supplementary material for: IRF1 ameliorates synaptic dysfunction through the modulation of O-GlcNAcylation on GluN1 subunit of NMDAR
Source: Alzheimers Res Ther. 2025 Sep 30;17:205. doi: 10.1186/s13195-025-01857-w (PMC12482151; doi:10.1186/s13195-025-01857-w)
Supplement: Supplementary file 1 — Supplementary Material 1 [file 13195_2025_1857_MOESM1_ESM.docx]

**Supplementary information for**

**IRF1** **ameliorates synaptic dysfunction through the modulation of O-GlcNAcylation on GluN1 subunit of NMDAR**

Xing Fan^1†^, Hao Wang^1†^, Cuiping Guo^2†^, Shijia Huang^5†^, Liye Xia^1^, Zheng Zhou^1^, Ran Tao^4^*, Mingzhe Li^3^*, Xiaochuan Wang^2^*, Wei Qian^1^*

This file includes:

Materials and Methods

Figs. S1 to S7

Table. S1

**Materials and Methods**

***Western blot analysis***: HEK-293T cells or animal brain tissues were lysed and assayed for protein concentration. Proteins were then separated by SDS-PAGE and transferred onto PVDF membranes (Millipore). The membranes were blocked with nonfat-dried milk and incubated with primary antibodies, followed by HRP-conjugated secondary antibodies. The target proteins were visualized using ECL luminescent solution (Merck) and a digital gel image-analysis system. Primary antibodies against IRF-1, OGA, OGT, β-actin, Flag, GluN1, and O-GlcNAc were obtained from Cell Signaling Technology. Antibodies for GAPDH and GluN2B were sourced from ProteinTech. The antibodies for GluN2A were from NeuroMab.

***Immunoprecipitation***: Brain hippocampal protein homogenates from IRF-1 knockout mice, 3xTg-AD mice injected with AAV-IRF-1 or AAV-OGA and corresponding littermate control mice were incubated overnight at 4 °C with protein A/G beads (Beyotime) conjugated with antibodies specific to subunits of NMADR (Cell Signaling Technology, NeuroMab, or ProteinTech). The next day, the precipitates were washed with an IP buffer, then added to 30 μl of 2xSDS sample buffer, and heated for 10 minutes at 95 °C. The O-GlcNAcylation levels of immunoprecipitated GluN1, GluN2A or GluN2B were detected and quantified using western blot analysis.

***Immunofluorescent staining***: Brain tissues from 2-3-month-old 3xTg-AD mice and their corresponding control mice were fixed with 4% paraformaldehyde, dehydrated in sucrose solution, and then embedded in OCT to collect 10 μm thick frozen sections. Afterwards, the sections were blocked with 5% BSA prepared in PBS containing 0.5% Triton X-100 for 1h at room temperature and incubated overnight with rabbit anti-IRF-1 antibody (Cell Signaling Technology). On the following day, the sections were blocked with TRITC-conjugated anti-rabbit IgG for 1h at room temperature, followed by a quench-resistant blocking agent containing DAPI, and then photographed.

***Quantification of mRNA***: Total RNA was extracted using Trizol (Beyotime), and 1μg of RNA was reverse transcribed with the 1st Strand cDNA Synthesis SuperMix for qPCR kit (Yeasen Biotech) and then detected by real-time PCR with SYBR Green Master Mix (Yeasen Biotech). Relative gene expression was normalized to GAPDH using the 2^-ΔΔCt^ method. The primers used were as follows: IRF-1, forward 5’-GTCCAGCCGAGATGCTAAGAG-3’ and reverse 5’-TGGTCATCAGGCAGAGTGGAG-3’; OGA, forward 5’-AGCCTTGAGTGGTGAG-3’ and reverse 5’- GTCATTCTTGTGGTCCG-3’; OGT, forward 5’-GAGTTCCGACTTTGGG-3’ and reverse 5’- CTGCCATCACCTTCACT-3’; GAPDH, forward 5’-GGCTACAGCAACAGGGTG-3’ and reverse 5’- TGGCAACTGTGAGGAGGG-3’.

***Chromatin immunoprecipitation***: After infecting HEK-293T cells with the shIRF-1 adenovirus for 48-72 h, chromatin was immunoprecipitated using ChIP Assay Kit (Beyotime) with an antibody against IRF-1 (Cell Signaling Technology). The IRF-1 binding to the *OGA* promoter region was then quantified and standardized using real-time PCR.

**Figure S1. Reduced distribution of IRF-1 in the hippocampus of 3xTg-AD mice. (A-B)** Immunofluorescence of IRF-1 in the hippocampal region of coronal brain sections from 3xTg-AD and WT mice (n=10). All data are presented as mean ± S.E.M. ****p*<0.001 vs. WT, two-tailed student’s *t* test.

**Figure S2. IRF-1 deficiency promotes O-GlcNAcylation of GluN1. (A)** Cortical tissue homogenates were collected from IRF-1 knockout (KO) mice to assess NMDARs expression through western blotting analysis (n=4). **(B-D)** The O-GlcNAcylation levels of immunoprecipitated GluN1 (B), GluN2A (C), or GluN2B (D) from brain homogenates of IRF-1 knockout (KO) mice were analyzed using western blots with anti-RL2, anti-GluN1, anti-GluN2A, or anti-GluN2B antibody. The relative O-GlcNAcylation levels (RL2) of immunoprecipitated GluN1, GluN2A or GluN2B were quantified after normalization with GluN1, GluN2A, and GluN2B (n=4). All data are presented as mean ± S.E.M. ***p*<0.01 vs. WT, two-tailed student’s *t* test or two-way ANOVA with Šídák's multiple comparisons test.

**Figure S3. IRF1 inhibits the O-GlcNAcylation of GluN1. (A-B)** The brain homogenates from 3xTg-AD mice injected with AAV-IRF-1 or its control vector were immunopurified using protein A/G beads conjugated with an anti-GluN1 antibody (A) or protein A/G beads conjugated with RL2 (B). The bound proteins were then subjected to western blot analysis with the antibodies indicated on each blot.

**Figure S4.** **IRF1 represses the O-GlcNAcylation of GluN1 via OGA.** pCDNA/GluN1 was transfected either alone or together with pCDNA/IRF1 into HEK-293T cells. After 24 h, HEK-293T cells were treated with 0.1, 1, 5, and 10 μm of Thiamet G for an additional 24 h. Then the cell lysates were immunoprecipitated with Protein A/G beads conjugated with the antibody against GluN1. The protein levels of GluN1 and the O-GlcNAcylation levels of proteins were examined using western blot analysis, employing antibodies as specified on each blot.

**Figure S5.** **The** **knockout of IRF-1 leads to a reduction in dendritic spine density.** **(A-C)** Representative images illustrating dendritic spines in hippocampal pyramidal neurons and their quantification. Dendritic spine density per 10 μm in hippocampal pyramidal neurons from IRF-1 knockout (KO) and WT mice were determined by Golgi staining. The percentages of stubby, mushroom, thin, or filopodium spines were also quantified (n=10). All data are presented as mean ± S.E.M. ***p*<0.01 vs. WT, two-tailed student’s *t* test or two-way ANOVA with Šídák's multiple comparisons test.

**Figure S6. IRF1 binds to the -332/-324 region of the OGA promoter to promote OGA expression transcriptionally.** **(A-C)** Correlation analysis revealed the relative levels of IRF-1 and OGA expression in all human brain samples (A) (n=20, R^2^=0.5704, P=0.0001), control brains (B) (n=10, R^2^=0.3940, P=0.0520), and AD brains (C) (n=10, R^2^=0.6478, P=0.0050) using Pearson analysis. **(D)** A positive correlation was observed between the relative levels of IRF-1 and OGA expression in all 3xTg-AD brain samples (n=6, R^2^=0.7064, P=0.0361) also through Pearson analysis. **(E)** Quantitative PCR confirmed the overexpression of IRF-1 in HEK-293T cells for OGA and OGT expression (n=3). **(F)** The binding of IRF-1 to the OGA promoter region at -332/-324 was examined using ChIP followed by real-time PCR with primers flanking this sequence (n=3). All data are presented as mean ± S.E.M. **p*<0.05; ***p*<0.01 vs. pcDNA, NC, two-tailed student’s *t* test or two-way ANOVA with Šídák's multiple comparisons test.

**Figure S7. OGA suppresses the O-GlcNAcylation of GluN1 and improves cognitive function in 3xTg-AD mice. (A-B)** The levels of OGA, OGT and O-GlcNAcylation in the brains of 3xTg-AD mice were determined by western blotting and quantitative analysis (n=12-13). **(C-E)** The O-GlcNAcylation levels of GluN1 immunoprecipitated from brain homogenates of 3xTg-AD mice injected with AAV-OGA were analyzed using western blots with RL2 and anti-GluN1 antibodies (C). The relative O-GlcNAcylation levels (RL2) of immunoprecipitated GluN1 were quantified after normalization with GluN1 (D) (n=6). The expression of OGA, GluN1 and O-GlcNAcylation in the brain tissues of 3xTg-AD mice injected with AAV-OGA was analyzed by western blotting (C) and quantitative analysis (E) (n=6). **(F)** A negative correlation was observed between the relative levels of OGA and the O-GlcNAcylation levels of immunoprecipitated GluN1 in the 3xTg-AD mice brain tissues (n=6, R^2^=0.6277, P=0.0021) (Pearson analysis). **(G)** 3xTg-AD mice injected with AAV-OGA were trained for 6 consecutive days to find platforms hidden in water, and escape latency was recorded and analyzed (n=10). **(H-I)** Swimming speed (m/s) and the times of platform crossing were recorded after the platform was removed on the 7th day (n=10-13). **(J)** Representative graphs of mice swimming trajectories during the testing phase. All data are presented as mean ± S.E.M. **p*<0.05; ****p*<0.001 vs. AAV-NC, two-tailed student’s t test or two-way ANOVA with Šídák's multiple comparisons test.

**Table S1.** **Human brain tissue from Alzheimer's disease (AD) and control (Con) cases used in this study**

| **Case** | **Age at death (years)** | | **Gender** | **PMI (hours)** | **Braak stage^*^** | **Tangle scores^#^** |
| --- | --- | --- | --- | --- | --- | --- |
| AD1 | 89 | F | | 3 | V | 14.5 |
| AD2 | 80 | F | | 2.25 | VI | 14.5 |
| AD3 | 85 | F | | 1.66 | V | 12 |
| AD4 | 78 | F | | 1.83 | VI | 15 |
| AD5 | 95 | F | | 3.16 | VI | 10 |
| AD6 | 86 | M | | 2.25 | VI | 13.5 |
| AD7 | 91 | F | | 3 | V | 8.5 |
| AD8 | 83 | F | | 3 | VI | 12.4 |
| AD9 | 74 | M | | 2.75 | VI | 14.66 |
| AD10 | 79 | F | | 1.5 | VI | 14.66 |
| Mean±SD | 84±6.48 |  | | 2.44±0.62 |  | 12.97±2.23 |
| Con1 | 85 | F | | 2.75 | II | 5 |
| Con2 | 82 | F | | 2 | II | 4.25 |
| Con3 | 70 | F | | 2 | I | 0 |
| Con4 | 73 | M | | 2 | III | 2.75 |
| Con5 | 78 | M | | 1.66 | I | 0 |
| Con6 | 80 | M | | 3.25 | I | 2.75 |
| Con7 | 80 | M | | 2.16 | II | 1 |
| Con8 | 83 | F | | 3.25 | I | 0.75 |
| Con9 | 82 | F | | 2.25 | II | 3.5 |
| Con10 | 85 | M | | 2.5 | II | 4.25 |
| Mean±SD | 79.80±4.94 |  | | 2.38±0.55 |  | 2.43±1.86 |

PMI: postmortem interval; * Neurofibrillary pathology was staged according to Braak and Braak (1995); ^#^Tangle score was a density estimate and was designated as none, sparse, moderate, or frequent (0, 1, 2, or 3 for statistics), as defined according to CERAD Alzheimer’s disease criteria.
